# Community-engaged analysis of soil lead contamination near a historical metallurgy facility in Los Angeles, California

**DOI:** 10.1007/s11356-025-37341-z

**Published:** 2026-01-12

**Authors:** Mason Thomas Reid, Wei-Cheng Hung, Eliza Lynch, Drew Ali, Zanobia Ibrahim-Watkins, Adriane Jones, Ambar Rivera, Kirsten Schwarz, Alexandra Temov, Rossmery Zayas, Jennifer Ayla Jay

**Affiliations:** 1 Samueli School of Engineering, University of California Los Angeles, 7400 Boelter Hall, Los Angeles, CA 90095 USA; 2 School of Environment and Natural Resources, The Ohio State University, 210 Kottman Hall, 2021 Coffey Rd, Columbus, OH 43210 USA; 3 Department of Chemistry, Southern Oregon University, 1250 Siskiyou Blvd, Ashland, OR 97520 USA; 4 Department of Environmental Studies, Wellesley College, 106 Central St, Wellesley, MA 02481 USA; 5 Department of Biological Sciences, Mount Saint Mary’s University, 12001 Chalon Road, Los Angeles, CA 90049 USA; 6 Communities for a Better Environment, 6325 Pacific Blvd. Ste 300, Huntington Park, CA 90255 USA

**Keywords:** Urban soils, Environmental justice, Lead characterization, Community-university partnerships, Metal toxicity

## Abstract

Legacy industrial lead (Pb) contamination poses a public health problem worldwide. In Southeast Los Angeles, California, a history of concentrated industrial activity has raised concern about Pb contamination in the area’s neighborhoods, particularly in Huntington Park. One former industrial facility in Huntington Park has attracted attention from both a local community environmental justice organization and the U.S. Environmental Protection Agency (USEPA). Although a 2023 report by the USEPA concluded that the Pb contamination from this site was “not significant,” the 2024 reduction of the federal soil screening level for residential soil Pb—from 400 mg/kg to 200 mg/kg—prompted a reassessment. In a community-based participatory research effort,91 residential surface soil samples from Huntington Park were analyzed by XRF spectrometry and compared with 118 surface soil samples from broader Los Angeles. In Huntington Park, 85.7% and 57.2% of soils exceeded the state (80 mg/kg) and new federal (200 mg/kg) screening levels, respectively—compared to 55.9% and 19.6% citywide. Soil Pb concentration was found to be statistically significantly higher in the residential soils in Huntington Park compared to the rest of Greater Los Angeles (Mann-Whitney *U*, *p* < 0.01). Soil Pb concentrations in Huntington Park also correlated significantly with proximity to the industrial site. Notably, 49.5% of Huntington Park soils fell between the updated and the former federal screening level, highlighting the need for reevaluation of the area’s contamination. These results also emphasize the need for remedial action to be taken in areas like Huntington Park, which serves as a clear example of disproportionate contaminant exposure faced by disadvantaged/minority communities.

**Supplementary Information:**

The online version contains supplementary material available at 10.1007/s11356-025-37341-z.

## Introduction

Exposure to heavy metal contamination is a major public health concern. Lead (Pb) is a heavy metal which is particularly well-researched due to its high toxicity, which can manifest even at low concentrations. In urban areas, the major pathways for human Pb exposure are inhalation and ingestion of soil particles and other dusts (Järup 2003; Ravipati et al. 2021; Wei an Yang 2010; Wani et al. 2015; Zahran et al. 2013). Pb is a naturally occurring constituent of soil, but anthropogenic sources such as lead-based paint, leaded gasoline, and industrial emissions can lead to elevated Pb levels in soils. Although Pb has largely been phased out of consumer products and industrial processes in the United States in recent decades, the high stability and low mobility of Pb in the environment have resulted in legacy pollution in soils where contaminant sources were once present (Mielke et al. 2010).

The toxicity of Pb is extremely well-documented and has been known by humanity since antiquity. The United States Agency for Toxic Substances and Disease Registry’s (ATSDR) latest comprehensive toxicological profile for Pb shows that Pb has been found to induce chronic neurological, renal, cardiovascular, hematological, immunological, reproductive, and developmental toxicity at the lowest studied concentrations of ≤ 5 μg/dL in blood (ATSDR 2020). Due to its extremely high potential for toxicity at even low doses, the ATSDR (2020) has declared that there is no safe level for Pb in blood, especially for children, whose developing systems and relatively lower mass make them more susceptible to toxicity. Studies in U.S. urban areas have shown an association between exposure to Pb-contaminated soil/dust and blood Pb levels in children (Johnson and Bretsch 2002; Mielke et al. 2007; Zahran et al. 2013). Further, research has indicated associations between Pb exposure in children and intellectual delays (Canfield et al. 2003; Koller et al. 2004), high school dropout rate (Needleman et al. 1990), and crime (Nevin 2007; Reyes 2007).

Los Angeles, California, with its history of heavy metal contamination from industrial activities and widespread car usage, has raised concerns about potential legacy Pb pollution. Previous studies have investigated Pb concentrations in surface soil across various land use types throughout the city. Clarke et al. (2015) and Wu et al. (2010) each showed that proximity to roadways and age of neighborhood were both factors associated with increasing concentrations of Pb in Los Angeles urban soils. Hung et al. (2018) found that soils in 35% of Los Angeles urban parks exceed the California residential soil screening level of 80 mg/kg (Office of Environmental Health Hazard Assessment 2009), which was also strongly correlated with the age of the park. Most recently, in Hung et al. (2023), soil Pb concentrations in Los Angeles residential yards were statistically significantly elevated compared to those observed at parks and schools. Findings in Los Angeles align with studies across the U.S. that have documented soil Pb patterns associated with housing age, distance to major roadways, distance to built structures (Schwarz et al. 2012), as well as land use (McClintock 2012).

Greater Los Angeles is known for its sprawling nature and consists of numerous incorporated and unincorporated communities. These different areas of Greater Los Angeles can have vastly different land use, demographics, and governance, which lends itself to a high stratification of the distribution of environmental contaminants. One prominent example of this phenomenon is the Exide battery recycling facility in Southeast Los Angeles’ Vernon, whose surroundings were investigated by both the state and federal government due to high levels of soil Pb and trichloroethylene in groundwater (USEPA 2024), attracting much media attention to the site. Recent scandals such as the listing on the Superfund National Priorities List (NPL) of Vernon’s Exide battery recycling facility have incited concern about metal-contamination exposure among nearby communities in Southeast Los Angeles (Aceves 2024), where much of the city’s industry is concentrated. Communities for a Better Environment (CBE), an environmental justice organization based in nearby Huntington Park, has highlighted the risks of heavy metal contamination in these industrial areas of Southeast Los Angeles for decades.

Community-based participatory research (CBPR) relies on meaningful collaboration between academic and non-academic partners to better address society’s challenges. A core principle is engagement of community members and researchers in the research process itself with the ultimate goals of enhanced relevance, broadened participation, and improved advancements in discovery (Balazs & Morello-Frosch 2013; Israel et al. 1998). Garcia et al. (2013) and Petersen et al. (2006) have applied CBPR in collaboration with communities in Greater Los Angeles to study air pollution, while Masri et al. (2020) partnered with a community group in Santa Ana to address soil Pb contamination. These collaborations demonstrate the effectiveness of CBPR in raising awareness of environmental issues and empowering communities to actively participate in creating positive change for unjustly polluted areas. In light of the USEPA’s screening of a local metallurgy site for the Superfund NPL followed by a reduction in federal soil screening levels for residential Pb on CERCLA and RCRA sites, the goals of this work were to incorporate these principles of CBPR to (1) quantify Pb in soils from residences surrounding this site in partnership with CBE, and (2) further characterize the spatial distribution of Pb contamination in the soils of Greater Los Angeles as a whole, using both existing and new data. Given previous investigation by the USEPA in Huntington Park’s possible contamination, it was hypothesized that residential soil Pb in this area would be elevated compared to the rest of Los Angeles.

This research effort also incorporated a course-based undergraduate research experience (CURE) component. CUREs are a type of inclusive pedagogy **(**Bangera and Brownwell 2014) that provide all enrolled students with access to applied research opportunities, which can improve retention for underrepresented groups (NASEM) and can increase confidence in applying technical skills (Callejas et al. 2023).

## Materials and methods

### Site characterization and history

The present work focused on investigating residential soil Pb surrounding the Central Metals Inc. site (hereafter CMI) in Southeast Los Angeles, a site of concern known to CBE and local residents. This site occupies a land area of 11.1 acres and is located at 33.96197° N, 118.23087° W. CMI operated an industrial metal recycling facility on the site from 2002 to 2016, although this plot of land has been home to industrial activity since the 1920 s (USEPA 2023). CMI’s operations included the cutting, shredding, and compacting of metal scraps for shipment. The facility stored scrap metal throughout the site in large debris piles that the USEPA (2023) noted were neither covered nor contained, something which is also attested by the residents of the area. Along with the advocacy of CBE, the concerns raised by the community about the operations of the facility led to the discovery of illegal and improper activities, including unpermitted operation and expansion. As a result, Los Angeles County did not grant a new permit to Central Metals Inc. in 2016, ending their operations. In the same year, the improper storage of hazardous waste also prompted an investigation by the USEPA.

In order to investigate a potential link between the CMI site and soil contamination in the area, the USEPA (2023) analyzed 249 soil samples from a depth of 10 cm with inductively coupled plasma-atomic emission spectrometry according to a sampling scheme based upon prevailing wind patterns. As a result, they sampled only homes in zones due East (Walnut Park neighborhood) and West (Florence-Firestone neighborhood) from the site. Because this approach restricted sampling to predefined zones, some households in Huntington Park—located immediately south of the site—were not sampled, even when adjacent properties to the east or west were included. This limited spatial coverage may help explain why the USEPA investigation found insufficient evidence to establish a connection between local soil contamination and CMI operations, citing a lack of correlations between soil Pb concentrations and distance from the site as well as low rates of exceedance of soil screening levels (USEPA 2023). Consequently, the site was not approved for listing on the Superfund NPL, although it remains a non-priority Superfund site.

### Analytical techniques

Portable X-Ray fluorescence spectrometry (pXRF) is a commonly used analytical technique of quantifying levels of Pb in soil, and its reliability in doing so is well supported. The levels of soil Pb were analytically determined by a Bruker S1 Titan portable X-ray fluorescence spectrometric analyzer according to USEPA SW-846 Test Method 6200 (2007). This method involved surface soil collection, drying, homogenization, insertion into specialized sample cups, and laboratory analysis by the XRF, the same procedure and equipment as described in Hung et al. (2023). Soil samples were collected at a depth of 0–5 cm as in Hung et al. (2023) between January and August of 2024. For both newly collected soil samples as well as those reported in Hung et al. (2018; 2023), XRF analysis was performed in triplicate and averaged.

### Sampling scheme

In contrast to the wind-based sampling scheme previously used by the USEPA, the sampling methodologies of the present work included community-engaged convenience sampling near the study site and a randomized grid approach for the comparison sites across Los Angeles. For sampling near CMI, community members present at CBE-hosted public forums and community meetings volunteered to have their soil tested, after which point 6–12 samples were taken from their yard to be analyzed separately ex situ, with the goal of sampling the widest variety of locations in the yard as possible. This would entail, for example, one site in the middle of the yard, one in the corner of the yard, one under a tree, one near a flower bed, and so on. The sample locations within the yard were also responsive to concerns of each home’s residents. Soil sampling was conducted across seven homes of public forum attendees. Without obtaining permission to test private residences, options for potential sampling sites were often much scarcer due to a lack of publicly accessible exposed earth in the heavily urbanized Huntington Park.

Ideally, the sites would be sampled in a regular, predetermined grid to better show spatial differences in metal concentrations—potentially a gradient of contamination over space. This is one of the sampling methods recommended by Davidson (2013) in her book on methods of soil heavy metal analysis, and undertaken by several studies similar to this one, including Schwarz et al. (2012), Urrutia-Goyes et al. (2018), and Hung et al. (2018). However, this prospect was unfeasible for the location surrounding CMI for several reasons. First, as mentioned, there is a major lack of public access to suitable sampling sites in the study neighborhood. The areas that are accessible to the public rarely possess any exposed soil at all, let alone sufficient areas of it that could be sampled in a grid. Another challenge to accessing suitable locations for soil sampling and analysis was posed by the fact that the vast majority of residents in the study neighborhood rent and do not own the homes in which they live. In soliciting services such as soil testing, renters often officially need approval from the property owners, who may have a vested interest in refusing it, especially if they suspect the results will show unsafe levels of contamination that may ultimately hurt their property value.

To provide context for the observations surrounding CMI, we added to existing data on soil Pb in the surrounding areas of Los Angeles. While Hung et al. (2023) tested soil samples for Pb in the laboratory from around Los Angeles County employing a randomized sampling scheme, there were some general regions of the county that were devoid of any sampling points. To determine where these supplemental samples should be collected, a grid with cell lengths of 5 km was overlaid on the map of samples collected by Hung et al. (2023), and any grid cells containing no points were flagged. Of those 88 cells that were not sampled by Hung et al. (2023), 10 cells were chosen at random, and fliers in English and Spanish were posted in collaboration with CBE calling for volunteers in these areas to have their soil tested (see Supplemental Information for flyer). Locations where fliers were posted include libraries, grocery stores, coffee shops, community centers, and parks. Forty-seven community members responded to the flyers, and of those, we were able to sample at 11 homes, with 15 more agreeing to mail their samples to the lab to be tested.

### Community-based methods

CBE has had longstanding involvement in research and advocacy on the CMI site and introduced researchers at the University of California, Los Angeles (UCLA) to the issue in 2023. In response to the new partnership between CBE and UCLA, CBE organized a community public forum, where UCLA researchers gave attendees a presentation responsive to community concerns about the routes of exposure of Pb and Arsenic (As) to humans, their toxicological effects, and ways to potentially prevent or lessen exposure. At this event, UCLA and CBE staff engaged with the community members and heard their personal concerns about the past, present, and future of the CMI site, which has since been purchased by a storage company and is pending redevelopment that would mandate demolition and construction, including heavy grading of contaminated soil. It is also at this event that community members could elect to sign up to have their soil tested for metals in the future. Once the soil of the community members had been analyzed, they received an individualized report detailing the results of their soil’s analysis and its implications.

As emphasized by Kerstetter (2012), engaging in community-based research entails reaching across racial, ethnic, and economic divides, among many other diverse demographic factors. This was reflected in our situation; the two census-designated places that made up the study area have very distinct demographics from many other areas of California, and even of Los Angeles itself. Florence-Firestone and Walnut Park are respectively 93.6% and 98.2% Hispanic or Latino, with only 14.5% and 11.4% of residents speaking English at home, respectively (U.S. Census Bureau, n.d.-a,  n.d.-c). Furthermore, these neighborhoods have higher rates of poverty than the City of Los Angeles at large, which itself is higher than the national rate. In addition, each neighborhood maintains a roughly 50% adult high school diploma attainment rate, compared to Los Angeles’s 78.7%, again, lower still than the national rate (U.S. Census Bureau, n.d.-b). Because of this community’s unique demographics and general distrust of outside bureaucracies stemming from historical exploitation of similar communities, it was crucial that a trusted, local entity bridge the gap that community members may perceive between external researchers and themselves, a principle discussed in London et al. (2018). As a familiar group of knowledgeable people well-established within the community, CBE facilitated this process, often even serving as or providing translators when needed in order for UCLA researchers to more effectively and respectfully engage with the community members. Throughout the collaboration, UCLA researchers employed reflective practices to recognize and respond to their positionality and power in the partnership with CBE and the local communities.

### Statistical analysis

To evaluate spatial patterns of Pb contamination in residential soils near the CMI site, we conducted a hotspot analysis using spatial autocorrelation methods. We applied the Getis-Ord Gi* statistic (Getis and Ord 1992) to detect statistically significant clusters of elevated Pb concentrations. This method assumes spatial dependence whereby sample points in proximity are more likely to exhibit similar values based on the hypothesis that Pb disperses gradually across space through environmental processes such as wind, water runoff, and soil movement. We used ArcGIS Pro to calculate Gi* z-scores and associated confidence intervals for each soil sample location. The analysis was performed using an inverse distance weighting (IDW) spatial relationship to account for the influence of neighboring samples. Hotspots (clusters of significantly high Pb values) were identified at 90%, 95%, and 99% confidence levels. Locations with z-scores greater than 1.65, 1.96, or 2.58 were interpreted as significant at the respective levels. This analysis allowed us to identify localized areas of concern, highlight spatial clustering near the CMI site, and assess potential links between environmental Pb levels and historical land use.

The residential soil Pb data were broadly grouped into two categories: Huntington Park and Greater Los Angeles. The Huntington Park dataset (*n* = 91) includes data collected at residences in and around Huntington Park throughout the first half of 2024. The residences tested for this dataset included only those of attendees of CBE community meetings who volunteered to have their soil tested. The Greater Los Angeles dataset (*n* = 118) consists of the residential soil Pb data reported in Hung et al. (2023) supplemented with the additional sampling described earlier. Of the 118 total Greater Los Angeles samples, 65 appear in Hung et al. (2023), and the remaining 53 were newly collected samples.

Since the data were not normally distributed, the difference in means between the residential soil Pb in the Huntington Park dataset and the Greater Los Angeles dataset was tested using the Mann-Whitney *U* test. To determine the distance between each soil sample location and the suspected polluter, one point in the middle of the CMI site was chosen, and the Euclidean distance was calculated between that point and each sample location. Linear and nonlinear regression were used to investigate relationships between soil Pb and distance from the suspected polluter. Due to the nature of the sampling scheme, many soil sampling locations in Huntington Park possessed nearly identical distances from the suspected polluter. Jenks clustering analysis was employed to account for this clustering of distance data (Jenks 1967).

## Results

### Distribution of residential soil Pb concentrations

 Within Huntington Park, the residential soil Pb values ranged from 38 to 5777 mg/kg with a median of 220 mg/kg and a mean of 285 mg/kg (SD = 600 mg/kg). In the rest of Greater Los Angeles, the residential soil Pb ranged from 0 to 837 mg/kg with a median of 81 mg/kg and a mean of 124 mg/kg (SD = 138 mg/kg). The highest soil Pb value obtained in Huntington Park was 5777 mg/kg, identified as a high outlier (the next highest recorded value was 1,069 mg/kg). While the high reading is thought not to be an analytical error due to consistent results of repeated analyses of the sample, it is unclear why this point was so much higher in Pb concentration, even relative to other nearby points on the same property. This value was excluded from some statistical analyses (e.g., regression modeling) to prevent it from exerting an undue influence on the statistical conclusions.

**Fig. 1:**
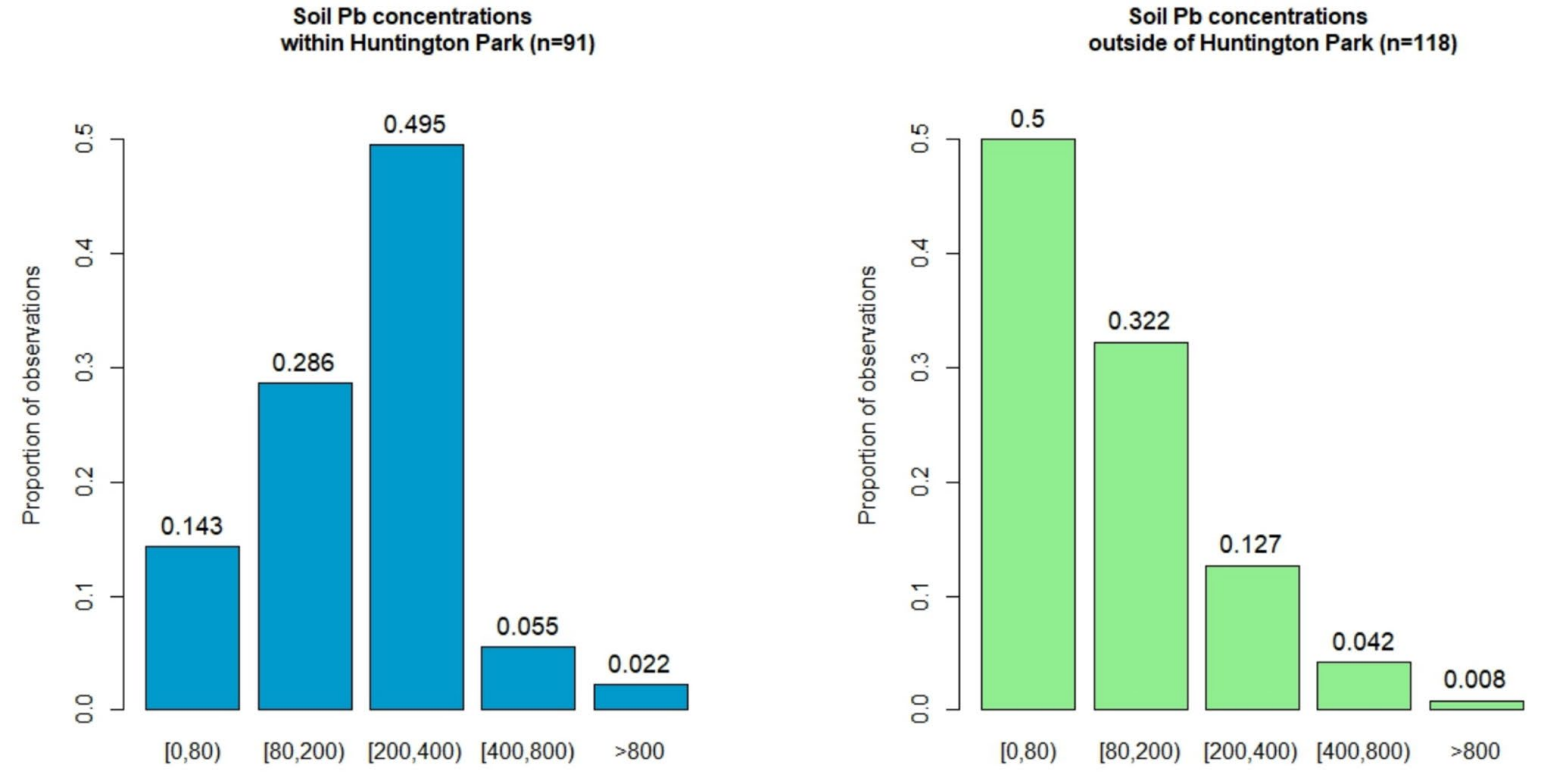
Statistical distribution of residential soil Pb inside Huntington Park (left) and in Greater Los Angeles (right) shown in modified histograms. The values atop each bar represent the proportion of observations within that bin

When using relevant values such as screening levels as bin thresholds, it is clear that the distribution of residential soil Pb in Huntington Park compared to Greater Los Angeles as a whole is starkly different (Fig. 1). In Greater Los Angeles, the vast majority of soils (> 80%) showed Pb concentrations below the current USEPA screening level of 200 mg/kg, with the plurality of soils (50.0%) showing Pb concentrations below even the stricter California state screening level of 80 mg/kg. Conversely, only 42.9% of soils sampled inside Huntington Park contained soil Pb levels below the current USEPA screening level, while only 14.3% of Huntington Park soils in total contained low enough Pb content to not exceed the California screening level. The proportion of residential soils below the screening level set by the State of California for Pb content is therefore about three times lower in Huntington Park than in the rest of Greater Los Angeles.

**Fig. 2:**
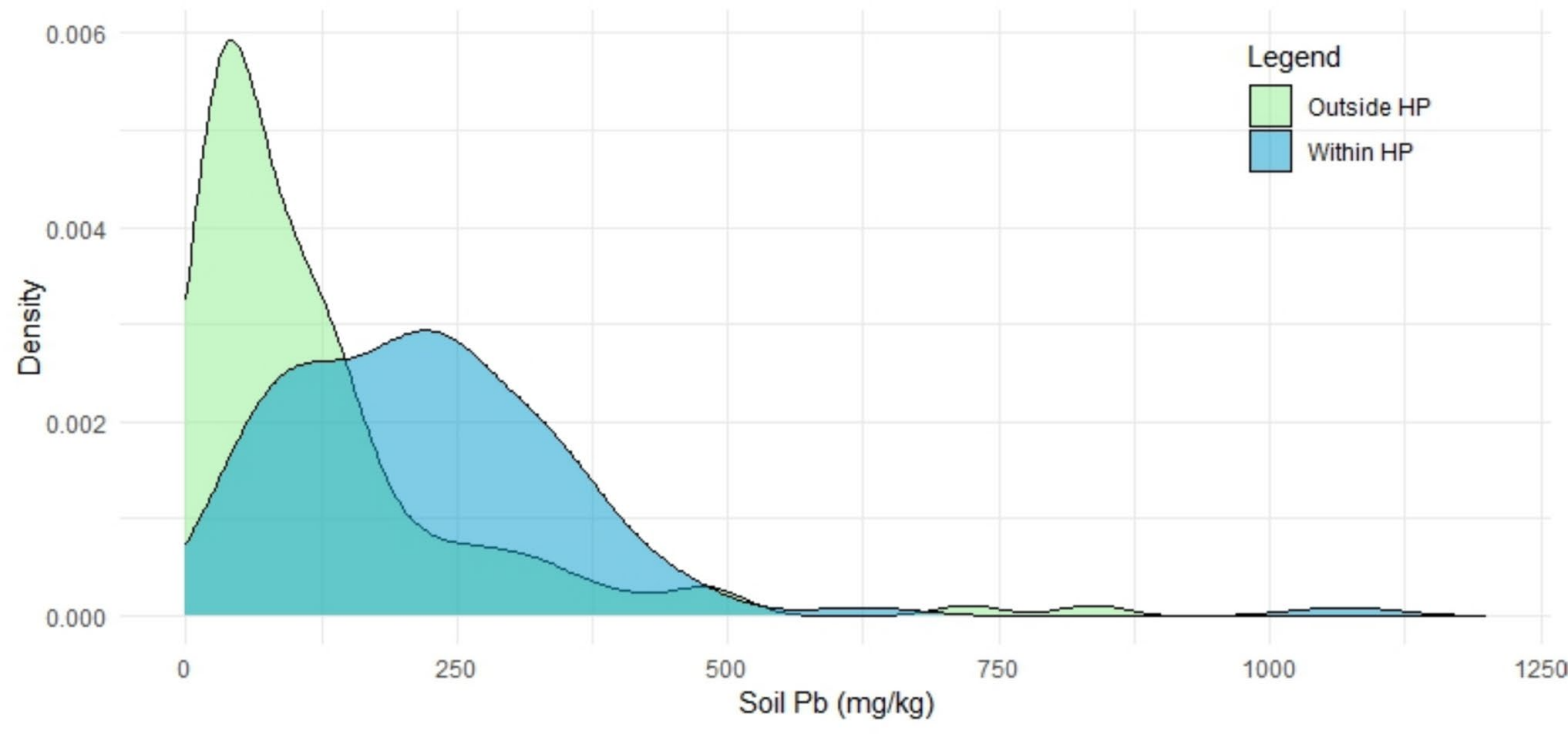
Density distribution plot of the soil Pb concentration within and outside Huntington Park. The view window is adjusted to showcase the majority of the data, but an outlier is excluded from view

The minimum, first quartile, median, third quartile, and maximum residential soil Pb detected in Huntington Park are all greater than those of Greater Los Angeles. In fact, the first quartile of residential soil Pb in Huntington Park is only marginally lower than the third quartile of residential soil Pb in Greater Los Angeles (115 and 152 mg/kg, respectively). Density distribution plots for the soil Pb concentrations from each of the two sample areas display this marked difference in Pb concentrations (Fig. 2) The Mann-Whitney *U* test confirms the suggestion of Fig. 2 that there is a difference in the distribution of the two datasets; there is a statistically significant difference in the residential soil Pb between Huntington Park and Greater Los Angeles as a whole among the soils sampled here (*p* < 0.01). This level of statistical significance is robust to the exclusion of the high outlier.

### Relationship between soil Pb and distance from suspected contamination source

**Fig. 3:**
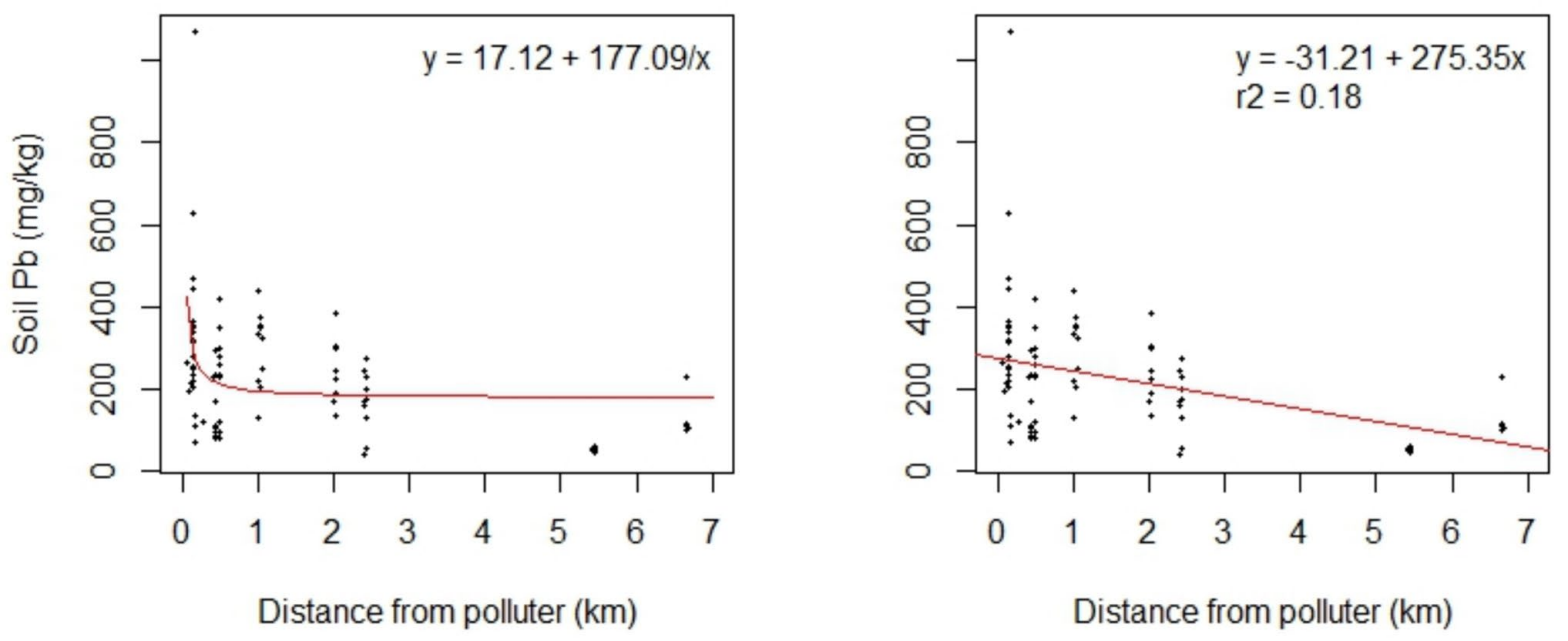
Scatterplots of Huntington Park soil Pb concentrations and distance from polluter. Nonlinear regression (left) and linear regression (right) are shown, both without the high outlier present

A statistically significant negative relationship between soil Pb and distance from the CMI site was observed when linear regression was performed (F-statistic = 18.8, *p* < 0.01) once the high soil Pb outlier was removed (Fig. 3). The r-squared value of this regression is low (*r*^2^ = 0.18), but the regression was statistically significant, showing that the samples closer to the site are generally more contaminated with Pb than those farther away. The root mean square error of this regression was 132.9, slightly better than the 137.9 of a nonlinear fit type, suggesting that the linear regression is the more appropriate model for this relationship. Regardless, the nonlinear regression resulted in statistical significance both with the outlier (F-statistic = 5.1, *p* < 0.05) and without the outlier (F-statistic = 12.3, *p *< 0.01). See Supplemental Information for the inclusion of the outlier.

**Fig. 4:**
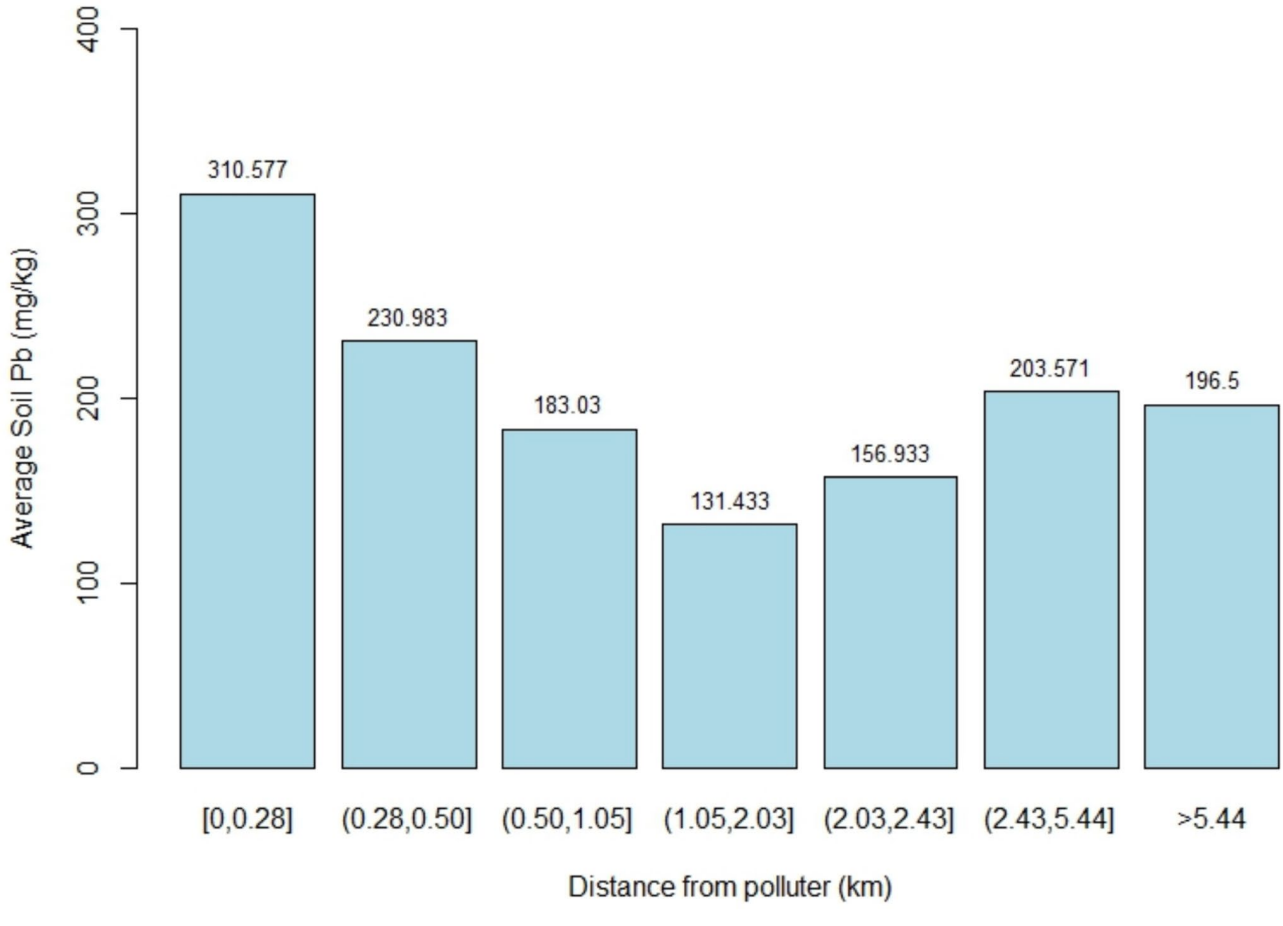
Huntington Park soil Pb with relation to distance from the suspected polluter. Bin thresholds were chosen according to results of Jenks clustering analysis

The sampling scheme resulted in multiple soil samples with nearly identical distances from the CMI site; these are samples collected from multiple areas on a single residential plot. To better show the distance/soil Pb relationship while accounting for this clustering, Jenks clustering analysis was performed to identify bin thresholds for a histogram relating distance with soil Pb (Fig. 4). While the statistically significant negative correlations observed with the linear and nonlinear regressions suggest that soil Pb decreases with increasing distance from the site, the clustering does not show a perfect sequential decrease in soil Pb as cluster distance increases. Instead, two clusters showed higher soil Pb than the immediately preceding cluster with less distance to the CMI site.

### Hot spot analysis

**Fig. 5:**
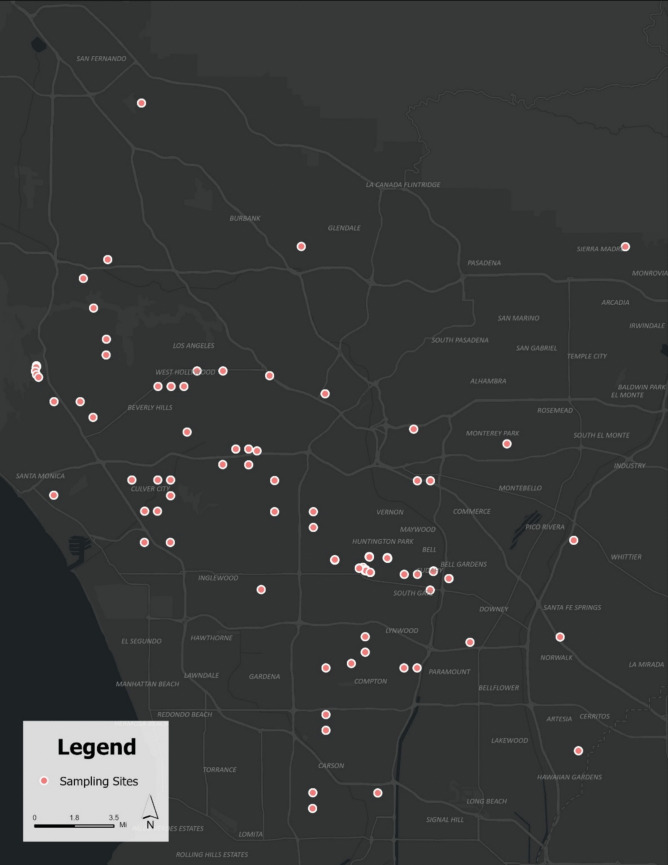
Sample locations throughout Los Angeles County and associated soil Pb values

Pb concentrations tend to be elevated near industrial areas, major roadways, or other localized sources of contamination. In this study, we assumed that Pb disperses gradually across space—via wind, water, or other environmental processes—such that nearby sample points exert greater influence on each other than those farther apart. The hotspot analysis identified statistically significant clusters of elevated Pb concentrations in residential soils surrounding the CMI site, with confidence levels of at least 90% (graph not shown). These clusters correspond with multiple previously observed elevated Pb concentrations in the area and indicate clear spatial clustering. Notably, the analysis identified a hotspot significant at the 99% confidence level, suggesting the presence of potential contamination sources. This hotspot is located near the intersection of Firestone Boulevard and Alameda Street, where Pb levels are substantially higher compared to surrounding areas. The soil Pb values associated with each sampled location throughout Greater Los Angeles including Huntington Park are displayed in Fig. 5.

## Discussion

### State of soil Pb contamination in Huntington Park

The most apparent finding of the present work is that residential soil in Huntington Park is widely contaminated with Pb beyond screening levels established by the USEPA (57.2% of samples) and especially the State of California (85.7% of samples). The results of soil testing in Greater Los Angeles suggest that the Pb contamination is an issue that is particularly localized to Huntington Park, with a statistically significant difference in residential soil Pb concentrations between Huntington Park and the rest of Los Angeles (Mann-Whitney *U*, *p* < 0.01). The statistical distribution of contamination detected in Huntington Park is particularly crucial. When the USEPA (2023) performed their investigation into CMI, their assessment and eventual rejection of the site’s placement on the Superfund NPL was based on the contemporaneous residential soil screening level of 400 mg/kg. Our finding that 7.7% of Huntington Park soil samples exceeded this threshold of 400 mg/kg is generally consistent with the USEPA’s (2023) own testing of Huntington Park’s residential soil, where 13% exceedance was detected. This 13% exceedance was one of the major factors that led the EPA to determine that contamination in the area was “minimal” (p. ES-2). After the USEPA study concluded, the federal residential soil screening level was reduced from 400 to 200 mg/kg. Critically, the state of contamination detected in Huntington Park by the present work showed that about one-half of soils were contaminated with Pb between the 200 and 400 mg/kg range. This finding implies that under the new federal residential Pb soil screening level, the exceedance in Huntington Park could be around 57% rather than 7–13%. It is unlikely that exceedances of 57% and 13% would lead to the same conclusion regarding future responses to the severity of contamination in the area; if the USEPA study were redone today, perhaps the decision whether to list the site on the Superfund NPL would be reconsidered. It is worth noting, however, that according to the average Pb in the USEPA (2023) residential shallow soil samples, only 24% exceeded the 200 mg/kg level. While this exceedance is not as high as the 57% detected by the present work, it is still just under twice as high as the exceedance reported using the pre-2024 federal soil screening level. The disparity in Pb contamination detected here and by the USEPA could be due to the depth of soil samples collected; the USEPA sampled down to four inches, while the soil collected here was not sampled as deeply—only 0–5 cm. In contaminated areas, soil Pb concentrations are highest at the surface, where atmospheric deposition occurs. A deeper soil sample is likely to incorporate a higher proportion of soil that has been exposed to less atmospheric deposition of Pb, potentially underestimating the level of associated Pb exposure. While the USEPA employed different analytical instrumentation than that used here, it is not likely that analytical differences alone would lead to a major difference in results, as both methods are supported by the literature in their ability to accurately quantify Pb in soil samples.

### State of soil Pb contamination in Los Angeles and California

Though the residential soil Pb was higher in Huntington Park than the rest of Los Angeles, the state of contamination detected in Los Angeles was elevated relative to state soil screening levels. The median residential soil Pb level of 81 mg/kg in Los Angeles (outside of Huntington Park) is itself slightly higher than the California screening level. In conjunction with the Huntington Park data, this suggests that residential soils in Los Angeles County in its entirety are elevated beyond the State of California screening level of 80 mg/kg. It is useful to compare these Pb levels from Los Angeles residential soils to those of California’s other major urban population center, the San Francisco Bay Area. Using ICP-AES as the analytical method, McClintock (2012) found the median residential soil Pb concentration in Oakland to be less than 50 mg/kg, and later in another study, McClintock (2015) corroborated this finding by recording a median soil Pb level of 59 mg/kg in Oakland. Both studies identify the West Oakland neighborhood of the Bay Area as a neighborhood where Pb levels are consistently higher, with the latter study reporting an average value of 573 mg/kg (median of 182 mg/kg) and a maximum value of 2262 mg/kg. McClintock (2012) attributes this trend to heavy historical industrial activity in the area, which parallels the history of Huntington Park. Regardless, the findings of these studies suggest that the concentrations of Pb in some San Francisco Bay Area residential soils may be lower than those of Los Angeles. This prospect is consistent with the findings of Mielke et al. (2010), which showed that Pb emissions in Los Angeles exceeded those of San Francisco during the late twentieth century. Further, the Oakland soil Pb levels reported by McClintock (2012; 2015) seem to be lower than the State of California’s screening level, while the Pb levels in Los Angeles reported by Hung et al. (2023) are generally much higher than this level. Though the analytical methods used here and by Hung et al. (2023) to quantify soil Pb differed from those used by McClintock (2012, 2015), the ability of XRF to reliably quantify soil contaminants is well supported, with Wu et al. (2012) showing that Pb is the single most reliably quantified element by XRF.

Given the findings shown here as well as by Hung et al. (2023) and McClintock (2012, 2015), it is clear that there is a need for the State of California and/or Los Angeles County to address the widespread Pb contamination of residential soils in Los Angeles, especially in Huntington Park, where the contamination is at its most severe. The gravity of this situation is further compounded by the fact that Los Angeles is the state’s most populous urban area, with a combined statistical area population over twice that of San Francisco, implying there is an enormous amount of people who could be potentially impacted by the consequences of this contamination.

### Impact of proximity to industry on soil Pb

The question on whether or not proximity to industry is related to the contamination in Huntington Park is one investigated both here and by the USEPA (2023), with the latter investigating specifically whether CMI was responsible for the area’s contamination. With a sampling scheme based on wind patterns, the USEPA sampled 83 residences directly East and West from the site and did not find strong evidence that contamination was higher closer to the site. In the present work, the sampling scheme was reliant on the willingness of the neighborhood residents to volunteer for soil sampling. It was predicted that capturing the contamination gradient from the suspected source would be more difficult with this sampling scheme; it was therefore expected that the evidence linking the contamination to CMI would be weaker relative to the USEPA study. Nevertheless, a statistically significant negative correlation between distance from the site and soil Pb was detected among the 91 soil samples across seven Huntington Park community members’ homes, as would be expected if the CMI site were responsible for Huntington Park’s contamination. As the distribution of contamination around the site is not thought to be uniform on all sides (the USEPA included atmospheric modeling in their sampling scheme for this reason), distance alone without respect for such complex transport and deposition processes should theoretically not show the relationship with soil Pb that is observed here. It is unclear why the atmospheric-based sampling scheme of the USEPA did not result in a similar correlation; it is possible that the heavy Pb-containing particulate matter was atmospherically deposited closer to the site than anticipated and in all directions from the site rather than only East and West due to changing wind direction. Another challenge to establishing a clear gradient of decreasing soil Pb with distance away from the site is the high level of industrialization in Southeast Los Angeles as a whole. For example, the CMI site is only about 6 km from the Exide battery recycling facility, which is currently nominated by the USEPA for listing on the Superfund NPL; the overlap of the “polluting influence” between these sites may obscure clean contaminant gradients that would be expected when establishing whether a particular site is the cause of a particular soil’s contamination. This phenomenon could explain why soil Pb seems to decrease sharply as distance from the CMI site increases only up until a point, after which it begins to slightly increase again, as shown in Fig. 4.

No matter the source, the results of the present work clearly demonstrate that there is significantly higher residential soil Pb in Huntington Park when compared to the rest of Los Angeles (Mann-Whitney *U*, *p* < 0.01), a finding further supported by hotspot analysis, which revealed statistically significant spatial clustering of elevated Pb concentrations near the suspected industrial source. This indicates a higher pollution burden faced by community members of Southeast Los Angeles, who are overwhelmingly Latino/Hispanic. The issue of environmental racism—environmental contamination disproportionately impacting minority communities—is a long-standing and widespread phenomenon in the United States (Cole and Foster 2001; Mohai et al. 2009; Wodtke et al. 2022; Taylor 2014). The results of the present work suggest that the situation in Huntington Park aligns with other environmental justice scholarship demonstrating that disadvantaged communities are disproportionately impacted by environmental harms. In addition, these results highlight Huntington Park as an area that calls for further environmental activism and future remedial intervention.

## Conclusion

The results of the analysis of residential soil in Huntington Park, Los Angeles, California show high levels of soil Pb. The majority of residential sites analyzed showed soil Pb levels above the screening level for Pb in residential soils established by the USEPA in 2024. Furthermore, only a small number of residential sites showed Pb levels below the screening level of the State of California. Comparing the levels of Pb detected in Huntington Park’s residential soil to that of the rest of Los Angeles County, it is apparent that Huntington Park has much higher levels of Pb than the rest of the county on average. While there is no definitive proof that the contamination in Huntington Park resulted from industrial activity on the CMI site, soil Pb was statistically significantly higher on average as the distance from the site decreased.

In collaboration with Communities for a Better Environment, the community members whose soils were tested over the course of the study were informed of the contamination detected in their yards, and of the implications of this contamination. The advocacy of CBE in Huntington Park concerning the future of the CMI site is ongoing, with the site currently pending redevelopment by a storage company. The community-based scientific effort addressing the soil Pb in Huntington Park will also continue alongside the advocacy work of CBE. With more data, it will be possible to obtain a greater understanding of the level of Pb contamination of Huntington Park’s soils, as well as how this compares to the level of Pb contamination present in the rest of Los Angeles. Ultimately, a clear picture of the highly contaminated soil in Huntington Park near the CMI site backed by strong, empirical evidence will show a strong need for action to enact change and protect the residents of Huntington Park from toxic impacts of soil contamination, regardless of the origin.

## Supplementary Information

Below is the link to the electronic supplementary material.

ESM 1 (PDF 235 KB)

ESM 2 (PDF 108 KB)

ESM 3 (PDF 97.8 KB)

## Data Availability

The data related to this study are included in the Supplemental Information. Longitude and latitude coordinates of the sample locations are not included to protect the privacy of individuals from whose property soil samples were included in the study.

## References

[CR1] Aceves M (2024) EPA Concurrence Request Letter to Governor Newsom Exide Vernon. https://dtsc.ca.gov/wp-content/uploads/sites/31/2024/07/EPA-Concurrence-Request-Letter-to-Governor-Newsom-Exide-Vernon_.pdf. Accessed 02 Sept 2024

[CR2] Balazs CL, Morello-Frosch R (2013) The three R’s: how community based participatory research strengthens the rigor, relevance and reach of science. Environ Justice (Print) 6(1). 10.1089/env.2012.0017 10.1089/env.2012.0017 PMC3832061 24260590

[CR3] Bangera G, Brownell SE (2014) Course-based undergraduate research experiences can make scientific research more inclusive. CBE—Life Sci Educ 13(4):602–606. 10.1187/cbe.14-06-0099 25452483 10.1187/cbe.14-06-0099 PMC4255347

[CR4] Callejas IA, Huang L, Cira M, Croze B, Lee CM, Cason T, Schiffler E, Soos C, Stainier P, Wang Z, Shaked S, McClellan M, Hung W-C, Jay JA (2023) Use of Google Earth engine for teaching coding and monitoring of environmental change: a case study among STEM and non-STEM students. Sustainability 15(15):11995. 10.3390/su151511995

[CR5] Canfield RL, Henderson Jr CR, Cory-Slechta DA, Cox C, Jusko TA, Lanphear BP (2003) Intellectual impairment in children with blood lead concentrations below 10 μg per deciliter.N Engl J Med 348(16):1517–1526. 10.1056/NEJMoa022848 10.1056/NEJMoa022848 PMC4046839 12700371

[CR6] Clarke LW, Jenerette GD, Bain DJ (2015) Urban legacies and soil management affect the concentration and speciation of trace metals in Los Angeles community garden soils. Environ Pollut 197:1–12. 10.1016/j.envpol.2014.11.015 25437835 10.1016/j.envpol.2014.11.015

[CR7] Cole LW, Foster SR (2001) From the ground up: environmental racism and the rise of the environmental justice movement. NYU Press

[CR8] Davidson CM (2013) Methods for the determination of heavy metals and metalloids in soils. In: Alloway BJ (ed) Heavy metals in soils: trace metals and metalloids in soils and their bioavailability. Springer, Netherlands, pp 97–140. 10.1007/978-94-007-4470-7_4

[CR9] Garcia AP, Wallerstein N, Hricko A, Marquez JN, Logan A, Nasser EG, Minkler M (2013) The (Trade, Health, Environment) impact project: a community-based participatory research environmental justice case study. Environ Justice 6(1):17–26. 10.1089/env.2012.0016

[CR10] Getis A, Ord JK (1992) The analysis of spatial association by use of distance statistics. Geogr Anal 24(3):189–206. 10.1111/j.1538-4632.1992.tb00261.x

[CR11] Hung W-C, Hernandez-Cira M, Jimenez K, Elston I, Jay JA (2018) Preliminary assessment of lead concentrations in topsoil of 100 parks in Los Angeles, California. Appl Geochem 99:13–21. 10.1016/j.apgeochem.2018.10.003

[CR12] Hung W-C, Adams N, Ibrahim-Watkins ZR, Nguyen D, Jain T, Wang Y-H, Jay JA (2023) Incorporating field-based research into remote learning: an assessment of soil lead pollution in different land-use types in Los Angeles. Environ Res 216:114480. 10.1016/j.envres.2022.114480 36206923 10.1016/j.envres.2022.114480

[CR13] Israel BA, Schulz AJ, Parker EA, Becker AB (1998) Review of community-based research: assessing partnership approaches to improve public health. Annu Rev Public Health 19(1):173–202. 10.1146/annurev.publhealth.19.1.173 9611617 10.1146/annurev.publhealth.19.1.173

[CR14] Järup L (2003) Hazards of heavy metal contamination. Br Med Bull 68(1):167–182. 10.1093/bmb/ldg032 14757716 10.1093/bmb/ldg032

[CR15] Jenks GF (1967) Data model concept in statistical mapping. Int Yearb Cartogr 7:186–190

[CR16] Johnson DL, Bretsch JK (2002) Soil lead and children’s blood lead levels in Syracuse, NY, USA. Environ Geochem Health 24(4):375–385. 10.1023/A:1020500504167

[CR17] Kerstetter K (2012) Insider, outsider, or somewhere between: the impact of researchers’ identities on the community-based research process. J Rural Soc Sci 27(2):7

[CR18] Koller K, Brown T, Spurgeon A, Levy L (2004) Recent developments in low-level lead exposure and intellectual impairment in children. Environ Health Perspect 112(9):987–994. 10.1289/ehp.6941 15198918 10.1289/ehp.6941 PMC1247191

[CR19] London JK, Schwarz K, Cadenasso ML, Cutts BB, Mason C, Lim J, Valenzuela-Garcia K, Smith H (2018) Weaving community-university research and action partnerships for environmental justice. Action Res 16(2):173–189. 10.1177/1476750316678915

[CR20] Masri S, LeBrón A, Logue M, Valencia E, Ruiz A, Reyes A, Lawrence JM, Wu J (2020) Social and spatial distribution of soil lead concentrations in the City of Santa Ana, California: implications for health inequities. Sci Total Environ 743:140764. 10.1016/j.scitotenv.2020.140764 32663692 10.1016/j.scitotenv.2020.140764 PMC7492407

[CR21] McClintock N (2012) Assessing soil lead contamination at multiple scales in Oakland, California: implications for urban agriculture and environmental justice. Appl Geogr 35(1–2):460–473. 10.1016/j.apgeog.2012.10.001

[CR22] McClintock N (2015) A critical physical geography of urban soil contamination. Geoforum 65:69–85. 10.1016/j.geoforum.2015.07.010

[CR23] Mielke HW, Gonzales CR, Powell E, Jartun M, Mielke PW (2007) Nonlinear association between soil lead and blood lead of children in metropolitan New Orleans, Louisiana: 2000–2005. Sci Total Environ 388(1):43–53. 10.1016/j.scitotenv.2007.08.012 17884147 10.1016/j.scitotenv.2007.08.012

[CR24] Mielke HW, Laidlaw MAS, Gonzales C (2010) Lead (Pb) legacy from vehicle traffic in eight California urbanized areas: continuing influence of lead dust on children’s health. Sci Total Environ 408(19):3965–3975. 10.1016/j.scitotenv.2010.05.017 20542539 10.1016/j.scitotenv.2010.05.017

[CR25] Mohai P, Lantz PM, Morenoff J, House JS, Mero RP (2009) Racial and socioeconomic disparities in residential proximity to polluting industrial facilities: evidence from the Americans’ Changing Lives Study. Am J Public Health 99(S3):S649–S656. 10.2105/AJPH.2007.131383 19890171 10.2105/AJPH.2007.131383 PMC2774179

[CR26] Needleman HL, Schell A, Bellinger D, Leviton A, Allred EN (1990) The long-term effects of exposure to low doses of lead in childhood. N Engl J Med 322(2):83–88. 10.1056/NEJM199001113220203 2294437 10.1056/NEJM199001113220203

[CR27] Nevin R (2007) Understanding international crime trends: the legacy of preschool lead exposure. Environ Res 104(3):315–336. 10.1016/j.envres.2007.02.008 17451672 10.1016/j.envres.2007.02.008

[CR28] Office of Environmental Health Hazard Assessment (2009) Revised California Human Health Screening Levels for Lead. https://oehha.ca.gov/media/downloads/crnr/leadchhsl091709.pdf. Accessed 24 Nov 2025

[CR29] Petersen D, Minkler M, Vásquez VB, Baden AC (2006) Community-based participatory research as a tool for policy change: a case study of the Southern California Environmental Justice Collaborative. Rev Policy Res 23(2):339–354. 10.1111/j.1541-1338.2006.00204.x

[CR30] Ravipati ES, Mahajan NN, Sharma S, Hatware KV, Patil K (2021) The toxicological effects of lead and its analytical trends: an update from 2000 to 2018. Crit Rev Anal Chem 51(1):87–102. 10.1080/10408347.2019.1678381 31650860 10.1080/10408347.2019.1678381

[CR31] Reyes JW (2007) Environmental policy as social policy? The impact of childhood lead exposure on crime. BE J Econ Anal Policy 7(1). 10.2202/1935-1682.1796

[CR32] Schwarz K, Pickett STA, Lathrop RG, Weathers KC, Pouyat RV, Cadenasso ML (2012) The effects of the urban built environment on the spatial distribution of lead in residential soils. Environ Pollut 163:32–39. 10.1016/j.envpol.2011.12.003 22325428 10.1016/j.envpol.2011.12.003

[CR33] Taylor DE (2014) Toxic communities: environmental racism, industrial pollution, and residential mobility. NYU Press

[CR34] United States Agency for Toxic Substances and Disease Registry (2020) Toxicological profile for lead. Agency for Toxic Substances and Disease Registry. 10.15620/cdc:95222

[CR35] United States Environmental Protection Agency (2007) SW-846 Test Method 6200: field portable X-ray fluorescence spectrometry for the determination of elemental concentrations in soil and sediment (6200). https://www.epa.gov/hw-sw846/sw-846-test-method-6200-field-portable-x-ray-fluorescence-spectrometry-determination. Accessed 10 Apr 2024

[CR36] United States Environmental Protection Agency (2023) Central Metal Huntington Park, Los Angeles County, California (Site Inspection Report No. CAN000903324). https://www.epa.gov/system/files/documents/2023-09/can000903324-central-metal-site-inspection-report-2023-09.pdf. Accessed 27 Mar 2024

[CR37] United States Environmental Protection Agency (2024) Exide Technologies—Vernon Vernon, Los Angeles County, California (Site Inspection Report No. CAN000903324). https://semspub.epa.gov/work/09/100037098.pdf. Accessed 15 Jan 2025

[CR38] Urrutia-Goyes R, Argyraki A, Ornelas-Soto N (2018) Characterization of soil contamination by lead around a former battery factory by applying an analytical hybrid method. Environ Monit Assess 190(7):429. 10.1007/s10661-018-6820-2 29946795 10.1007/s10661-018-6820-2

[CR39] U.S. Census Bureau (n.d.-a) QuickFacts: Florence-Graham CDP, California; Lafayette city, California; Compton city, California. https://www.census.gov/quickfacts/fact/table/florencegrahamcdpcalifornia,lafayettecitycalifornia,comptoncitycalifornia/PST045222. Accessed 24 Apr 2024

[CR40] U.S. Census Bureau. (n.d.-b) QuickFacts: Los Angeles city, California. https://www.census.gov/quickfacts/fact/table/losangelescitycalifornia/PST045223. Accessed 24 Apr 2024

[CR41] U.S. Census Bureau (n.d.-c) QuickFacts: Walnut Park CDP, California; Carson city, California. https://www.census.gov/quickfacts/fact/table/walnutparkcdpcalifornia,carsoncitycalifornia/BZA115221. Accessed 24 Apr 2024

[CR42] Wani AL, Ara A, Usmani JA (2015) Lead toxicity: a review. Interdiscip Toxicol 8(2):55–64. 10.1515/intox-2015-0009 27486361 10.1515/intox-2015-0009 PMC4961898

[CR43] Wei B, Yang L (2010) A review of heavy metal contaminations in urban soils, urban road dusts and agricultural soils from China. Microchem J 94(2):99–107. 10.1016/j.microc.2009.09.014

[CR44] Wodtke GT, Ard K, Bullock C, White K, Priem B (2022) Concentrated poverty, ambient air pollution, and child cognitive development. Sci Adv 8(48):1–19. 10.1126/sciadv.add0285 10.1126/sciadv.add0285 PMC9710878 36449613

[CR45] Wu J, Edwards R, He X(, Liu Z, Kleinman M (2010) Spatial analysis of bioavailable soil lead concentrations in Los Angeles, California. Environ Res 110(4):309–317. 10.1016/j.envres.2010.02.004 20219189 10.1016/j.envres.2010.02.004

[CR46] Wu C-M, Tsai H-T, Yang K-H, Wen J-C (2012) How reliable is X-ray fluorescence (XRF) measurement for different metals in soil contamination? Environ Forensics 13(2):110–121. 10.1080/15275922.2012.676603

[CR47] Zahran S, Laidlaw MAS, McElmurry SP, Filippelli GM, Taylor M (2013) Linking source and effect: resuspended soil lead, air lead, and children’s blood lead levels in Detroit, Michigan. Environ Sci Technol 47(6):2839–2845. 10.1021/es303854c 23428083 10.1021/es303854c

