# Supplementary material for: Community-engaged analysis of soil lead contamination near a historical metallurgy facility in Los Angeles, California
Source: Environ Sci Pollut Res Int. 2026 Jan 12;33(2):637–48. doi: 10.1007/s11356-025-37341-z (PMC12882941; doi:10.1007/s11356-025-37341-z)
Supplement: Supplementary file 1 — (PDF 235 KB) [file 11356_2025_37341_MOESM1_ESM.pdf]

Supplementary Information for:

Community-engaged, course-based analysis of soil lead contamination near a historical metallurgy facility in Los Angeles, California

Authors: Mason Thomas Reid[1,2], Wei-Cheng Hung[3*], Eliza Lynch[2,4*], Drew Ali[2], Zanobia Ibrahim-Watkins[2], Adriane Jones[5], Ambar Rivera[6], Alexandra Temov[2], Rossmery Zayas[6], Kirsten Schwarz[2], Jennifer Ayla Jay[2]

*** These two authors contributed equally to this work.**

1. The Ohio State University, School of Environment and Natural Resources, 210 Kottman Hall, 2021 Coffey Rd, Columbus, OH 43210
2. University of California Los Angeles, Samueli School of Engineering, 7400 Boelter Hall
Los Angeles, CA 90095
3. Southern Oregon University, Department of Chemistry, 1250 Siskiyou Blvd, Ashland, OR 97520
4. Wellesley College, Department of Environmental Studies, 106 Central St, Wellesley, MA, 02481
5. Mount Saint Mary's University, Department of Biological Sciences, 12001 Chalon Road Los Angeles, CA 90049
6. Communities for a Better Environment, 6325 Pacific Blvd. Ste 300; Huntington Park, CA 90255

Corresponding author: Jennifer Jay, jennyjay@ucla.edu, cell: 310-866-2444, fax: 310-206-2222

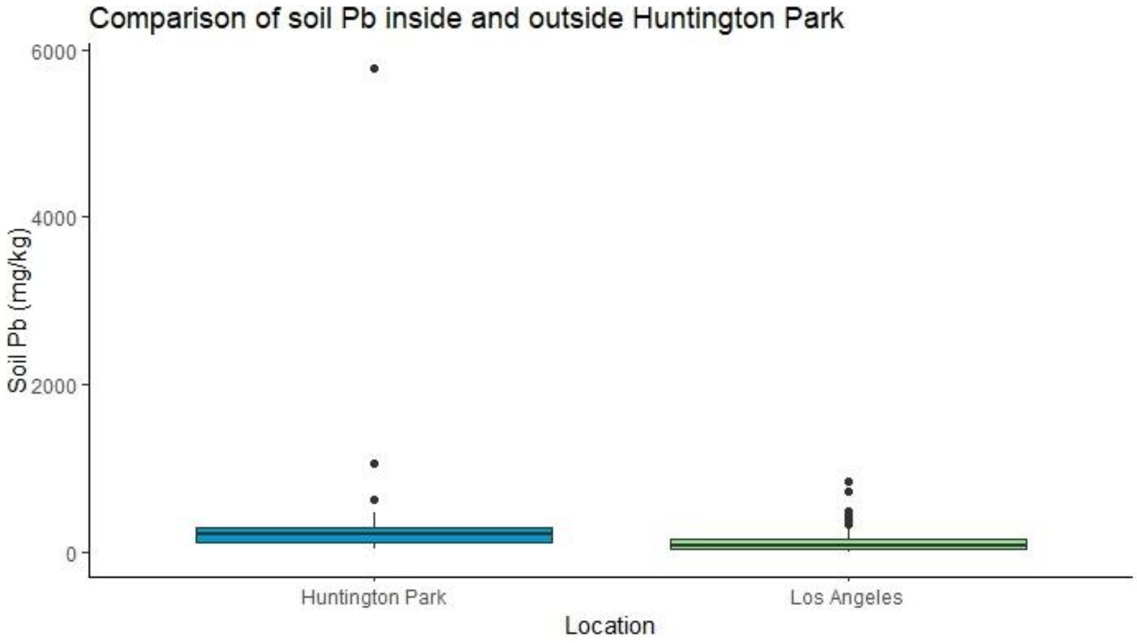

SI 1. Boxplots of soil-Pb for inside and outside of Huntington Park with visible high outlier

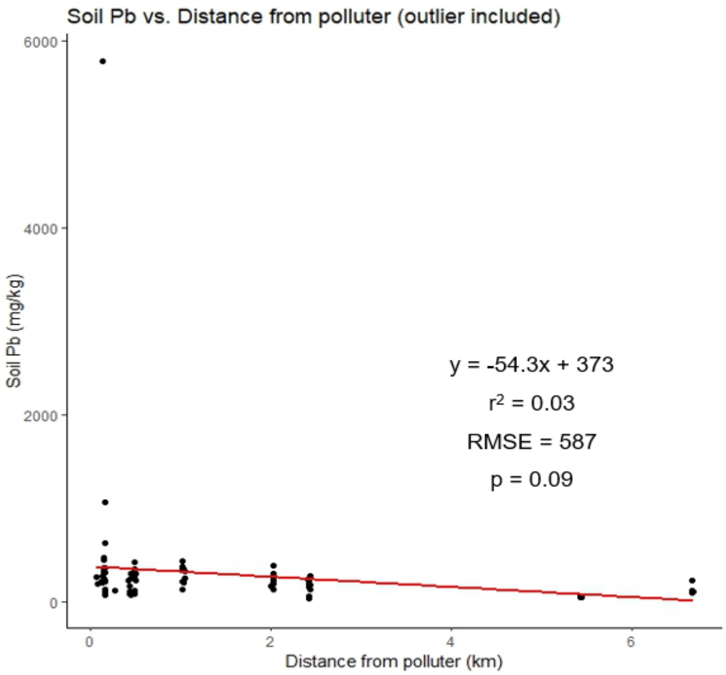

SI 2. Scatterplot of Huntington Park soil-Pb and Distance from polluter including linear regression with high outlier present

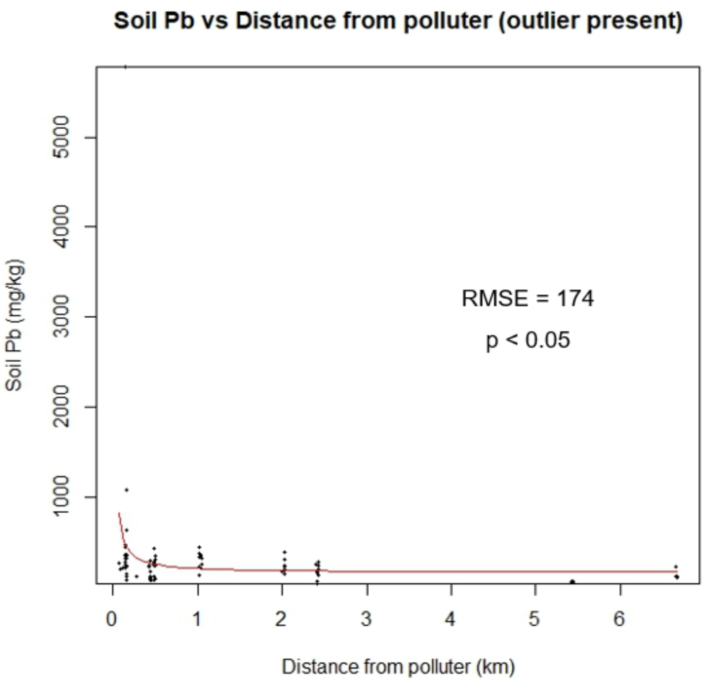

SI 3. Scatterplot of Huntington Park soil-Pb and Distance from polluter including nonlinear regression with high outlier present
