# Supplementary material for: Community-engaged analysis of soil lead contamination near a historical metallurgy facility in Los Angeles, California
Source: Environ Sci Pollut Res Int. 2026 Jan 12;33(2):637–48. doi: 10.1007/s11356-025-37341-z (PMC12882941; doi:10.1007/s11356-025-37341-z)
Supplement: Supplementary file 2 — (PDF 108 KB) [file 11356_2025_37341_MOESM2_ESM.pdf]

¿Le preocupa que los contaminantes del suelo dañen a su familia?

GRATIS PRUEBAS DE SUELO

Communities for Better Environment (CBE) y UCLA se están asociando para brindar a todos los residentes pruebas de suelo gratuitas. El análisis proporcionaría a los residentes conocimiento sobre la presencia de metales tóxicos, particularmente plomo, en el suelo que rodea sus hogares.

Para obtener más información y compartir su interés en participar, complete el "Formulario de interés en pruebas de suelos" hoy.

Formulario de interés:

https://forms.gle/8W5uutcznxAfjDwg8

UCLA Samueli School of Engineering

COMMUNITIES FOR A BETTER ENVIRONMENT
40 years | established 1978
