# Supplementary material for: Community-engaged analysis of soil lead contamination near a historical metallurgy facility in Los Angeles, California
Source: Environ Sci Pollut Res Int. 2026 Jan 12;33(2):637–48. doi: 10.1007/s11356-025-37341-z (PMC12882941; doi:10.1007/s11356-025-37341-z)
Supplement: Supplementary file 3 — (PDF 97.8 KB) [file 11356_2025_37341_MOESM3_ESM.pdf]

Worried about soil pollutants harming your family?

FREE SOIL TESTING

Communities for Better Environment (CBE) and UCLA are partnering to provide all residents with free soil testing. The analysis would provide residents with the knowledge of the presence of toxic metals, particularly lead, in the soil surrounding your homes.

Interest Form

To learn more & share your interest in participating, complete the "Soil Testing Interest Form" today!

https://forms.gle/5CbgvAm7iyDvN8Dw7

UCLA Samueli School of Engineering

COMMUNITIES FOR A BETTER ENVIRONMENT
40 years | established 1978
